# Suppressing H19 Modulates Tumorigenicity and Stemness in U251 and U87MG Glioma Cells

**DOI:** 10.1007/s10571-015-0320-5

**Published:** 2016-03-16

**Authors:** Weiguo Li, Pengfei Jiang, Xiaoling Sun, Shujun Xu, Xiangyu Ma, Rucai Zhan

**Affiliations:** 1 Neurosurgery Department, Qilu Hospital, Shandong University, 107 Wenhua West Road, Lixia District, Jinan, 250012 Shandong China; 2 Yantai Yuhuangding Hospital, Yantai, China; 3 Neurosurgery Department of No. 3 Hospital of Jinan, Jinan, China

**Keywords:** LncRNA, H19, Glioblastoma, Glioma stem-like cells

## Abstract

Glioblastoma multiforme (GBM) is a type of malignant carcinoma found in the brain. Its high frequency of occurrence and poor survival rate have garnered much research attention in recent years. Long non-coding RNAs (lncRNAs) are known to be related to the formation and progression of several cancer types by both promoting and suppressing tumor transformation. H19 is one such lncRNA and has been shown to be upregulated in a few types of cancer. In this study, we discovered that the expression of H19 increased in GBM cell lines. H19 knocked down GBM cells also displayed decreased cellular proliferation and a higher apoptosis rate when induced by temozolomide. Interestingly, the GBM cell lines U87MG and U251 were found to express cancer stem cell markers CD133, NANOG, Oct4 and Sox2. Expression of these markers was downregulated in H19-deficient cells. Collectively, these data suggest a role for H19 in contributing to GBM malignancy and the maintenance of its stem cell properties.

## Introduction

Glioblastoma multiforme (GBM), or glioblastoma, is a type of brain tumor arising from astrocytes, the star-shaped glial cells that support and protect neural tissues in the brain and spinal cord (Furnari et al. 2007; Perry et al. 2004; Rosenblum 2007). Due to the extensive network of blood vessels in the brain, GBM cells proliferate rapidly, making GMB the most aggressive form of primary brain tumor (DeAngelis 2001; Maes and Van Gool 2011; Wrensch et al. 2002). GBM is the most prevalent brain tumor found in adults (DeAngelis 2001), and patients diagnosed with GBM usually have a poor prognosis; the median survival time is one year under standard treatment (Yan et al. 2013). Treatment typically involves surgery followed by radiotherapy. The addition of chemotherapy via temozolomide (TMZ) was shown to increase patients’ survival period (Reardon et al. 2006; Stupp et al. 2005). However, the appearance of radio/chemotherapy-resistant GBM cells easily invalidates this treatment (Ahmed et al. 2013; Rich and Eyler 2008). Recent progress in cancer biology has identified that the resistant cells are mainly from a group expressing stem cell markers, which are defined as cancer stem cells (CSCs), or cancer-initiating cells (Clevers 2011; Lobo et al. 2007). CSCs are tumorigenic: a small population of CSCs is able to give rise to a tumor with heterogeneous cell types (Clevers 2011; Frank et al. 2010; Lobo et al. 2007; Price et al. 2008). In the particular case of GBM, these have been referred to as glioma stem cells (GSCs) (Frosina 2011; Ye et al. 2012). Although great effort has been made to study GSCs, their role in GBM remains largely unknown. Therefore, more research is necessary to identify the mechanisms that cause GBM and its reoccurrence in order for treatments to advance.

One of the most promising targets in GBM treatment has been the discovery of the role of long non-coding RNA (lncRNA). Non-coding RNA is transcribed from non-coding DNA, which represents around 98 % of human DNA and was initially considered “junk DNA” (International Human Genome Sequencing et al. 2001). LncRNA is defined as non-coding RNA of over 200 nucleotides in length (Gupta et al. 2010; Gutschner and Diederichs 2012; Zhu et al. 2011). Research has recently revealed the critical roles of lncRNAs in tumorigenesis and the progression of a few types of cancer, including GBM. Numerous lncRNAs have been identified at present (Kiang et al. 2015). Wang et al. reported that one lncRNA, colorectal neoplasia differentially expressed (CRNDE), was upregulated in glioma patients and cell lines. CRNDE promotes the proliferation and invasion of glioma cells by modulating the mTOR-signaling pathway (Wang et al. 2015). Yao et al. showed that another lncRNA, X-inactive specific transcript (XIST), was also upregulated in glioma tissues and GSCs. The authors demonstrated the role of XIST in tumorigenesis by showing that the XIST-knockdown glioma cells displayed inhibited tumor growth, migration and invasion, and reduced apoptosis by regulating miR-152 (Yao et al. 2015).

Although a number of lncRNAs have been discovered that contribute to the formation and progression of GBM, the effort to look for differentially expressed lncRNAs in GBM continues. In the past 10 years, four independent groups have reported upregulation of H19 in GBM cells and GSCs. H19 was shown to encode an RNA transcript that was highly expressed in mouse embryos, and was later determined to be involved in cell proliferation in both mouse and human models (Bartolomei et al. 1991; Feil et al. 1994; Pachnis et al. 1984). Further studies demonstrated its involvement in GBM progression and invasion (Shi et al. 2013, 2014). However, the mechanisms involved remain unclear.

Our goal in this study was to examine the function of H19 in GBM as well as the underlying molecular mechanisms. To do this, we selected GBM cell lines and confirmed H19 upregulation in these cells. H19 was knocked down in several GBM cell lines, after which the cell proliferation rate, apoptosis, tumor formation capability, and expression of CSC markers were measured. We successfully identified several oncogenic functions of H19.

## Materials and Methods

### Cell Culture

Human glioblastoma cell lines U87MG, U251, U343, Hs683, LN215 and A172, and normal human astrocytes (NHA) cell line were cultured in Dulbecco’s modified Eagle’s medium (DMEM) (Invitrogen Life Technologies, USA), supplemented with 10 % fetal bovine serum (Gbico, USA), 100 U/ml penicillin, and 100 μg/ml streptomycin. All cells were incubated in a humidified atmosphere containing 5 % CO_2_ at 37 °C.

### SiRNA Transfection

Knockdown of H19 in all GBM cell lines was established via RNAi using a lentiviral vector. SiRNA oligonucleotides and the lentivirus were purchased from Ribo Biotech (Guangzhou, China). RT-PCR was performed after transduction to confirm the knockdown efficiency. SiRNA powders were dissolved in nuclease-free water to give a 50 μM stock concentration and then diluted to a 10 nM working concentration for all transfections. The ribonucleic acid sequences of siRNAs targeting H19 were as follows (5′–3′): sense, GCGGGUCUGUUUCUUUACUUU, antisense, AGUAAAGAAACAGACCCGCUU. The ribonucleic acid sequences of control siRNA were as follows (5′–3′): sense, GCGUUCUGGUCUUACUGUUUU, antisense, AGAGAAUAAACCCGCAGACUU. Transfection and co-transfection of siRNA were performed according to the manufacturer’s protocol of lipofectamine 2000 (Invitrogen).

### RNA Extraction and RT-PCR

The RNeasy Mini Kit (Qiagen) was used to extract the total RNA. RT-PCR was performed on an ABI PRISM 7500 (Applied Biosystems) with SYBR^®^*Premix Ex Taq*™ (Perfect Real Time) (Takara). The oligonucleotide sequences used for RT-PCR analyses are shown in Table 1.

**Table 1:** Primers

Gene	Primer	Product size (bp)
Variant 1	Sense: GGCAAGAAGCGGGTCTGT	273
	Anti-sense: GCTGCTGTTCCGATGGTGT	
Variant 2	Sense: GGCTCTGGAAGCTAGAGGAA	168
	Anti-sense: CTGGGATGATGTGGTGGC	
Variant 3	Sense: GACCCAAGGACTCAAGCG	115
	Anti-sense: GCGAGACTCCAGGAACACT	
GAPDH	Sense: TGTGGGCATCAATGGATTTGG	116
	Anti-sense: ACACCATGTATTCCGGGTCAAT	
CD133	Sense: AGTCGGAAACTGGCAGATAGC	99
	Anti-sense: GGTAGTGTTGTACTGGGCCAAT	
Nanog	Sense: TTTGTGGGCCTGAAGAAAACT	116
	Anti-sense: AGGGCTGTCCTGAATAAGCAG	
Oct-04	Sense: GTGTTCAGCCAAAAGACCATCT	156
	Anti-sense: GGCCTGCATGAGGGTTTCT	
Sox2	Sense: TACAGCATGTCCTACTCGCAG	110
	Anti-sense: GAGGAAGAGGTAACCACAGGG	

### Western Blot Analysis

Cells were washed in ice cold phosphate-buffered saline (PBS) and then treated with a RIPA protein lysis buffer to prepare protein lysates as previously described (Pierce et al. 1999). The protein concentrations in the cell lysates were measured by Bio-Rad protein assay kits (Bio-Rad, Hercules, CA, USA) and then calibrated by standard bovine serum albumin concentrations. 40 μg total proteins for each cell lysate sample were loaded and separated by SDS-PAGE on a 10 % gel, and transferred to PVDF membranes (Bio-Rad). 5 % bovine serum albumin in Tris buffer (TBS) was used for membrane blocking overnight at 4 °C, and the membrane was then washed with PBS containing 0.1 % Tween 20 (TBST). Primary antibodies specific to target proteins were used for probing for 1 h at room temperature and then washed with TBST again; corresponding secondary antibodies were used for detection. Finally, the membrane was subjected to autoradiography, and signals were quantified. Primary antibodies (mouse monoclonal) specific to CD133 and OCT4 were purchased from MACS Miltenyi Biotec; NANOG antibody (rabbit polyclonal) was purchased from Santa Cruz Biotech; SOX2 antibody (goat polyclonal) was purchased from Santa Cruz Biotech; GAPDH antibody (mouse monoclonal) was purchased from Zhongshanjinqiao Biotech. Secondary antibodies were purchased from Zhongshanjinqiao Biotech. Film autoradiography reagents were purchased from Bio-Rad.

### CCK-8 Assay Analysis

The CCK-8 solution was used to quantify the cell proliferation rate. Cells seeded in a 96-well plate were treated with 10 µl CCK-8 solution, and incubated for 2 h at 37 °C. The automated ELISA reader (Bio-Tek Instruments Inc., Winooski, VT, USA) was used to quantify absorbance (*A*) of each well at 450 nm, and the cell survival rate (%) was then calculated as follows:

$$[A({\text{experiment}}) - A({\text{blank}})]/[A({\text{control}}) - A({\text{blank}})]\,*\,100.$$

### EdU Incorporation Assay

EdU assay kit (Ribobio, Guangzhou China) was used to detect the proliferation status according to the manufacturers’ instructions. Cells were briefly cultured in triplicate in confocal dishes at a density of 2 × 10^3^ cells for 48 h at 37 °C, and 50 µM of EdU was then added to each well. Cells were then cultured for an additional 2 h at 37 °C. The cells were then fixed with 4 % formaldehyde for 15 min at room temperature and treated with 0.5 % Triton X-100 for 20 min at room temperature for permeabilization. After washing with PBS three times, 100 µl of a 1× Apollo^®^ reaction cocktail was added to each well and the cells were incubated for 30 min at room temperature. The cells were then stained with 100 µl of Hoechst33342 for 30 min and visualized under a fluorescent microscope (Olympus Corporation, Tokyo, Japan).

### Flow Cytometry Analysis

An Annexin V-PE apoptosis kit (BD Biosciences, Franklin Lakes, NJ, USA) was used to measure apoptosis. U87MG and U251 cells were first transfected with si-H19 or negative controls respectively, and the cells were then harvested and washed with ice cold PBS. The washed cells were re-suspended in an Annexin V binding buffer, followed by staining with Annexin V-PE and 7-AAD. The fluorescence signals of stained cells were then read by FACS (Becton-Dickinson, Franklin Lakes, NJ, USA), and the results were analyzed using Cell Quest software (Becton-Dickinson).

### Transwell Assay

The invasion assay was performed using a Transwell chamber (Corning, NY, USA). 1 × 10^5^ cells in serum-free medium were placed into the upper chamber with an insert coated with Matrigel (BD Biosciences, San Jose, CA, USA). Next, medium containing 10 % FBS was added to the lower chamber. After 24 h of incubation, the cells remained on the upper membrane were eliminated, and the cells invaded through the membrane were fixed and stained with methanol and 0.1 % crystal violet. The cells were imaged using an IX71 inverted microscope (Olympus, Tokyo, Japan).

### Statistical Analysis

All statistical analyses were performed with an unpaired Student’s *t* test. A *p* < 0.05 was considered statistically significant.

## Results

### Glioblastoma (GBM) Cells Showed Upregulated H19 Expression

**Fig. 1:**
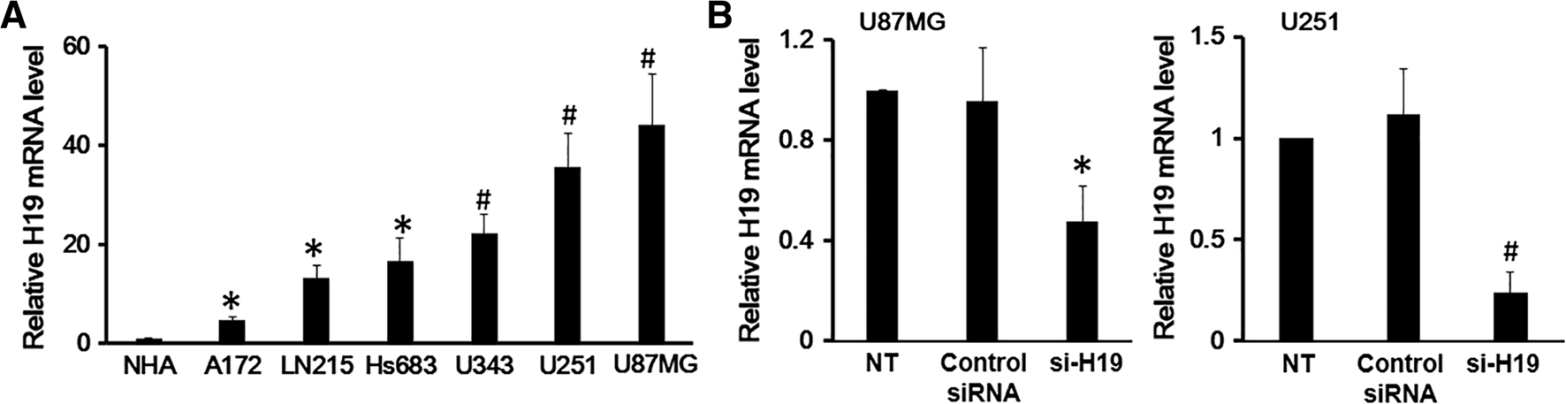
Glioblastoma (GBM) cell lines showed upregulated H19 expression. **a** The lysates of GBM cells from A172, LN215, Hs683, U343, U251 and U87MG cell lines were harvested and examined for H19 expression; the cell lysates of Normal Human Astrocytes (NHA) cell line were used as the negative control. **b** The efficiency of H19 knockdown by siRNA in U87MG and U251 cells was measured by qRT-PCR. Non-treated (NT) U87MG and U251 cells and cells transfected with control siRNA were included as controls. Data here represent an average of three independent experiments; the *error bar* indicates the standard deviation of the mean; *p < 0.05; ^#^ *p* < 0.005

Since GBM cells are derived from normal astrocytes by malignant transformation, we first compared H19 expression in GBM cells and NHAs. To do this, H19 expression was measured in six different GBM cell lines: U87MG, U251, U343, Hs683, LN215 and A172. H19 expression in GBM cell lines was significantly higher than in the NHA cell line (Fig. 1a). Among the 6 GBM cell lines, U87MG cells showed a 44-fold upregulation of H19 expression compared to NHA cells, and U251 cells showed a 35-fold upregulation. Therefore, U87MG and U251 cell lines were chosen as representative GBM cell lines for the subsequent studies.

Next, we wanted to explore the function of H19 in GBM cells. To do this, H19 was knocked down by si-RNA in U87MG and U251 cell lines. The results indicated a 50 % knockdown efficiency in U87MG and 70 % efficiency in U251 (Fig. 1b).

### H19 Knockdown Reduced the Proliferation Rate and Migration Activity of GBM Cells

**Fig. 2:**
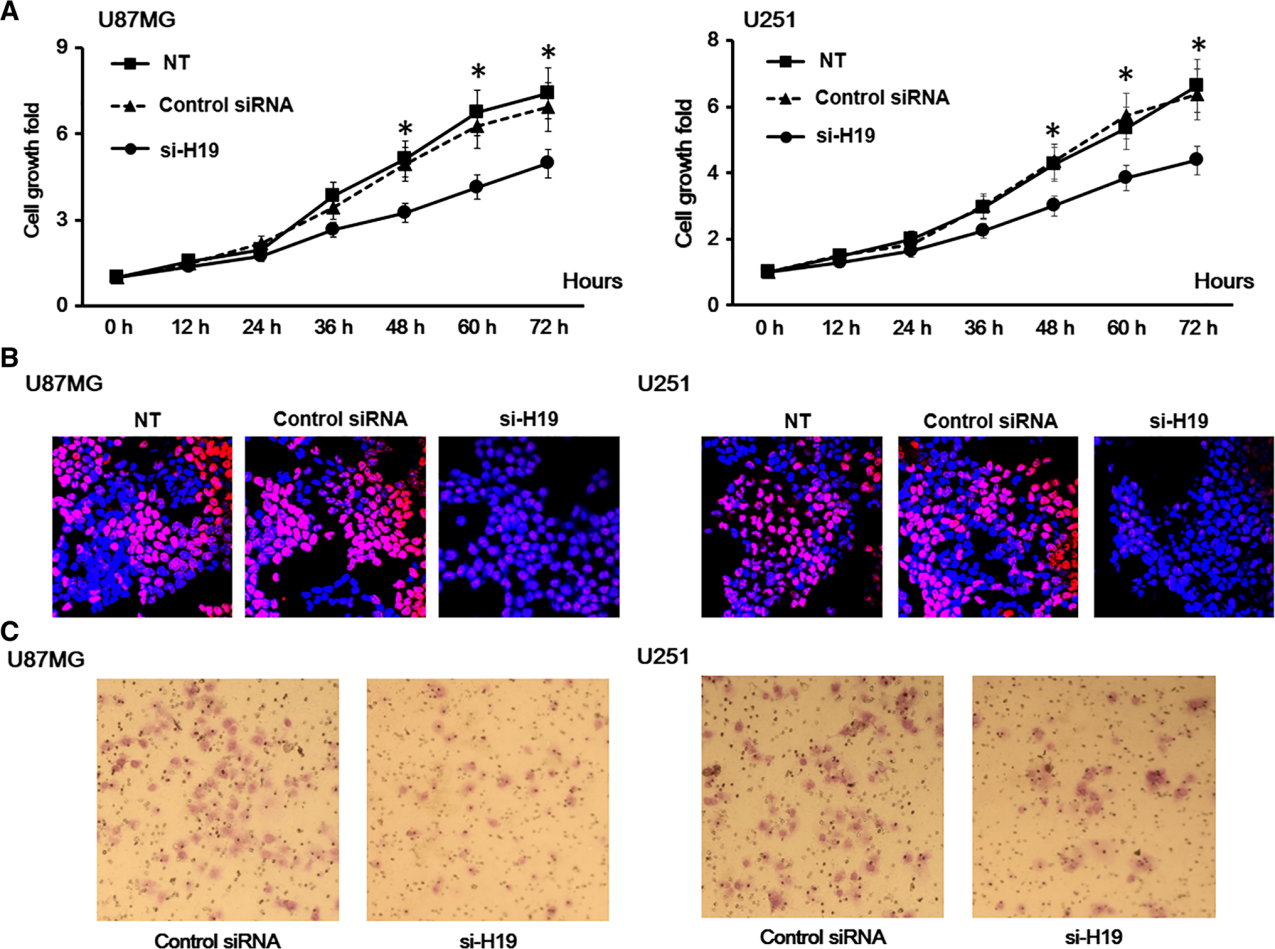
H19 knockdown decreased the proliferation rate of glioblastoma (GBM) cells. **a** The proliferation of U87MG and U251 cells with H19 knockdown was measured by CCK-8 assay. U87MG and U251 cells transfected with non-specific control siRNA and no siRNA (NT) were both included as negative controls. Data here represent an average of three independent experiments; *error bar* indicates standard deviation of the mean; *p < 0.05; ^#^ *p* < 0.005. **b** The proliferation of U87MG and U251 cells with H19 knockdown was measured by EdU assay. U87MG and U251 cells transfected with non-specific control siRNA and no siRNA (NT) were both included as negative controls. Three independent experiments were done; representative fluorescent microscope images were shown here. **c** The invasion of U87MG and U251 cells with H19 knockdown was measured by Transwell assay. U87MG and U251 cells transfected with non-specific control siRNA and no siRNA (NT) were both included as negative controls. Five pictures were collected for each group, and the representative images were shown here

By knocking down H19 in U87MG and U251 cell lines, we found that they both displayed decreased proliferation rates compared to non-treated cells and cells transfected with a non-specific control siRNA (Fig. 2a). Edu staining also confirmed a reduced proliferation rate in H19 knockdown GBM cells (Fig. 2b). These results indicate a role of H19 in the proliferation of GBM cells.

Next, we studied if H19 knockdown would also affect cell migration and invasion in GBM cell lines. We found that both U87MG and U251 cells displayed reduced cell mobility after H19 knockdown (Fig. 2c).

### H19 Knockdown Led to Increased TMZ-Induced Apoptosis in GBM Cells

**Fig. 3:**
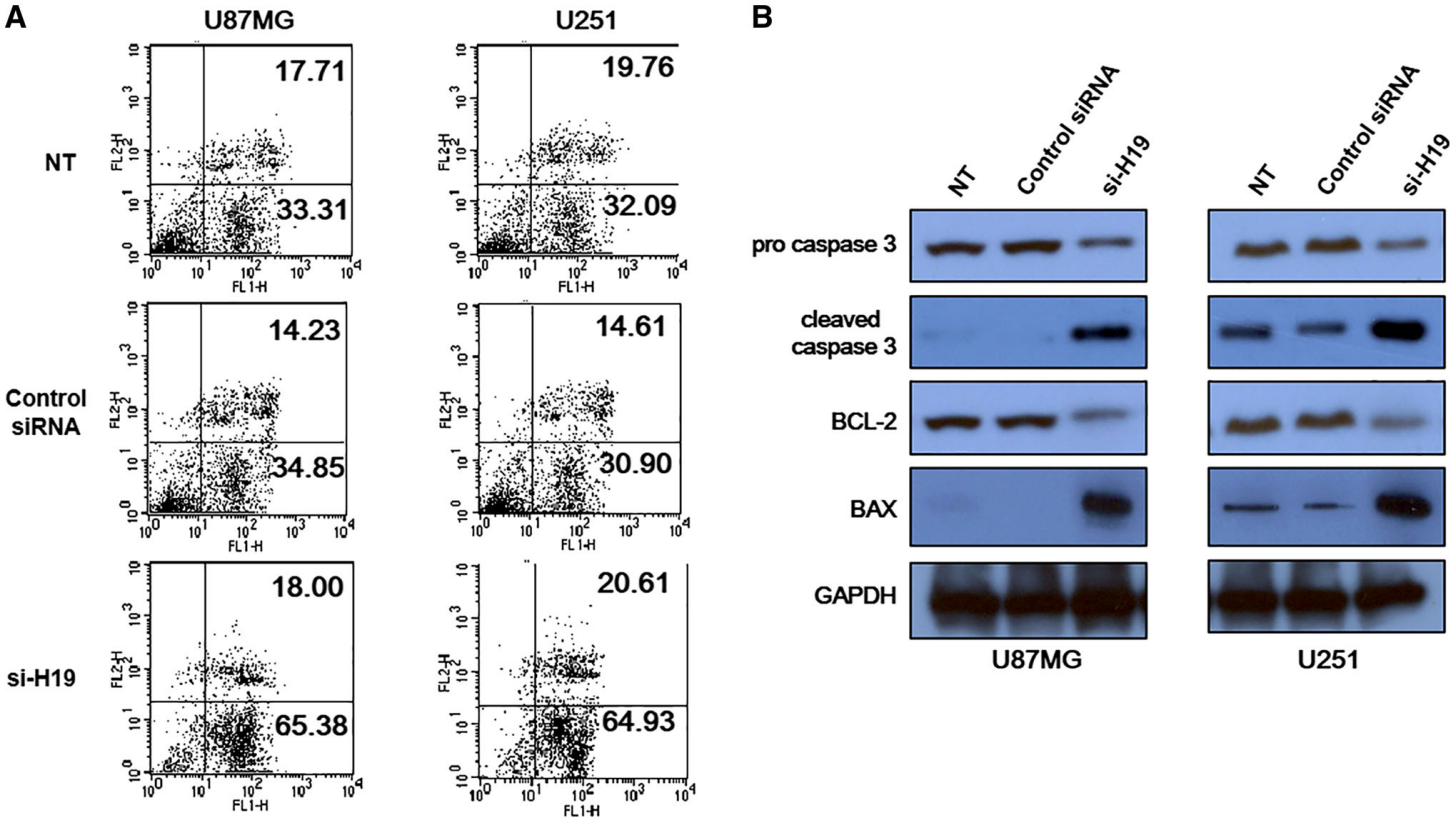
H19 knockdown led to apoptosis in glioblastoma (GBM) cells. **a** Apoptosis of U87MG and U251 cells with H19 knockdown was measured by Annexin V-FITC/PI double staining assay. TMZ-treated U87MG and U251 cells transfected with non-specific control siRNA and non-treated (NT) were both included as negative controls. Three independent experiments were done; representative flow cytometry data were shown here. **b** Protein expression level of pro caspase 3, cleaved caspase 3, BCL-2 and BAX was measured in U87MG and U251 cells with H19 knockdown. U87MG and U251 cells transfected with non-specific control siRNA and no siRNA (NT) were both included as negative controls

After determining that H19 reduced the proliferation rate of GBM cells, we wondered whether it would also increase apoptosis. To address this question, we induced apoptosis with temozolomide (TMZ), a widely used chemotherapeutic medicine to treat glioblastoma (He et al. 2015). The function of H19 in TMZ-induced GBM cell apoptosis was determined by comparing H19-knockdown GBM cells with normal GBM cells. The results showed that TMZ-treated U87MG and U251 cells with H19 knockdown had a larger proportion undergoing apoptosis compared to non-treated cells or TMZ-treated cells transfected with non-specific control siRNA (Fig. 3a). Most of the apoptotic cells were found in the early stages, as indicated by Annexin V positive and PI negative staining (Fig. 3a). Cells with H19 knockdown had a slightly higher number in necrosis, indicated by double positive staining, compared to the negative controls (Fig. 3a). This result suggested the involvement of H19 in the anti-apoptosis process of GBM cells.

Since H19-knocked down GBM cells showed signs of apoptosis in staining, we sought to confirm this by measuring the level of metabolic markers in apoptosis. Our results showed that TMZ-induced U87MG and U251 cells with H19 knockdown had reduced pro-caspase 3 and elevated cleaved caspase 3 expression compared to the negative controls. Similarly, H19 knockdown caused increased expression of Bax, and decreased the Bcl-2 level in TMZ-induced U87MG and U251 cells (Fig. 3b). These data confirmed that H19 knockdown led to increased apoptosis of GBM cells under TMZ treatment.

### Cancer Stem Cell Markers were Downregulated in GBM Cells with H19 Knockdown

**Fig. 4:**
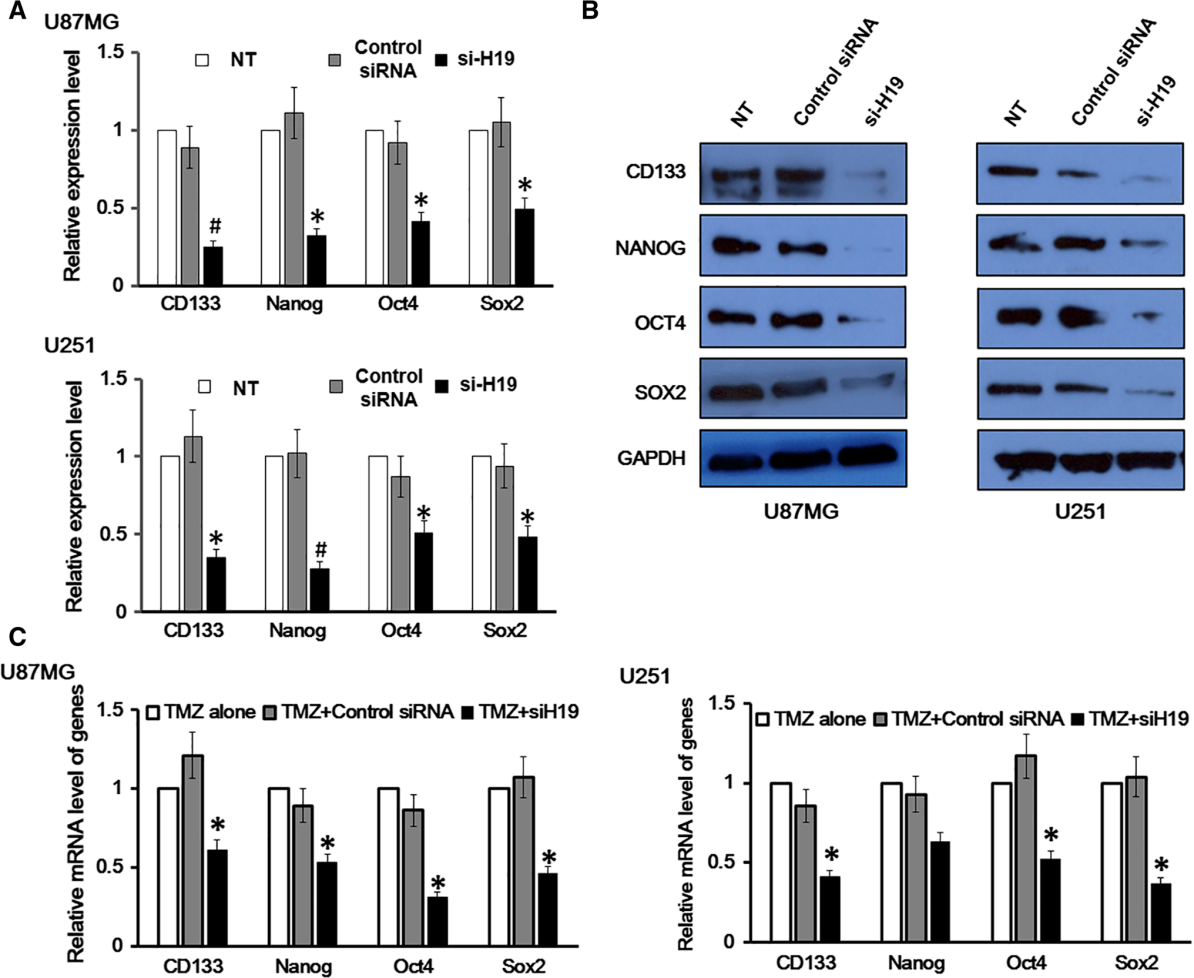
Cancer stem cell (GSC) markers were downregulated in glioblastoma (GBM) cells with H19 knockdown. **a** mRNA level of CD133, Nanog, Oct-4 and Sox2 was measured in U87MG and U251 cells with H19 knockdown. U87MG and U251 cells transfected with non-specific control siRNA and no siRNA (NT) were both included as negative controls. Data here represent an average of three independent experiments; *error bar* indicates standard deviation of the mean; *p < 0.05; ^#^ *p* < 0.005. **b** Protein level of CD133, Nanog, Oct-4 and Sox2 was measured in U87MG and U251 cells with H19 knockdown. U87MG and U251 cells transfected with non-specific control siRNA and no siRNA (NT) were both included as negative controls. **c** mRNA level of CD133, Nanog, Oct-4 and Sox2 was measured in U87MG and U251 cells treated with temozolomide (TMZ). TMZ-alone and TMZ + control siRNA groups were both included as negative controls. Data here represent an average of three independent experiments; *error bar* indicates standard deviation of the mean; *p < 0.05

We have so far shown that H19 knockdown impaired the proliferation of GBM cells and also increased their apoptosis under TMC induction. By examining H19 knockdown cells, we found that they adopted a rounded shape and became less attached, which led us to wonder whether they harbor stem cell properties. To investigate this, both the mRNA and protein levels of four stem cell markers were measured in U87MG and U251 cells, and they were all found to be positive (Fig. 4a, b). However, H19 knockdown in these cells reduced the expression of the four markers in both mRNA (Fig. 4a) and protein levels (Fig. 4b). These findings suggest that the oncogenic properties of H19 may be due to transforming differentiated astrocytes into GSCs.

Next, we examined whether TMZ would also affect stem cell marker expression in GMB cell lines. To do this, we divided both U87MG and U251 cells into three groups. The first group was treated with TMZ alone; the second was transfected with control siRNA and treated with TMZ; and the third was transfected with H19 siRNA and treated with TMZ. We found that TMZ treatment alone and TMZ-treated cells transfected with control siRNA showed a very similar expression level of the four stem cell markers, while TMZ-treated cells transfected with H19 siRNA showed significantly reduced expression, which was about 40–60 % of the expression in the other two conditions (Fig. 4c).

## Discussion

Using representative cell lines, we examined the role of H19 in GBM. We found that H19 promoted cell proliferation in GBM since U87MG and U251 GBM cells with H19 knockdown exhibited a reduced cell proliferation rate (Fig. 2). In addition, we showed that TMZ-induced apoptosis increased in U87MG and U251 GBM cells with H19 knockdown (Fig. 3), which suggested the anti-apoptosis function of H19 in GBM. Finally, a screening of stem cell markers found that their expression dropped significantly in H19-deficient GBM cells (Fig. 4), indicating the involvement of H19 in the maintenance of the GSC population.

Our study successfully established a correlation between H19 and the proliferation of GBM cells. H19 has been known for its involvement in cell proliferation in mouse embryos since shortly after its discovery three decades ago (Pachnis et al. 1984). Later research identified H19 in human cells and found a close link with insulin-like growth factor 2 (Igf2) via their reciprocal imprinting in embryos (Feil et al. 1994; Zhang and Tycko 1992). However, these imprinting studies revealed that H19 functioned to downregulate cellular proliferation (Bartolomei et al. 1991; Feil et al. 1994), which was contradictory to our discovery in GBM cell lines. Similarly, the function of H19 in cancer is also in debate. Previous studies have shown that H19 bears both oncogenic (Adriaenssens et al. 1998; Moulton et al. 1994) and tumor-suppressive properties (Hao et al. 1993; Yoshimizu et al. 2008). However, recent research has supported the former role by demonstrating upregulation of H19 in a few types of cancer and its involvement in promoting cancer invasion, migration and metastasis (Huang et al. 2015; Liu et al. 2015; Yang et al. 2015; Zhou et al. 2015). Here we found that H19 was upregulated in GBM cells, especially those in a late-stage, since the U87MG cell line originated from a stage-IV GBM patient. These findings support the oncogenic function of H19 in tumor formation and development. However, it is worth noting that these seemingly contradictory roles of H19 were found in different types of cancer. It is likely that H19 plays different roles in different tissues or developmental stages, and its role in a specific tissue remains the same in both normal and tumor cells. For example, H19 was shown to repress cellular growth in embryos and was also found to be a tumor suppresser in embryonic carcinoma (Hao et al. 1993).

Besides mediating cell proliferation, H19 was also found to be responsible for anti-apoptosis in GBM cells in this study. We found that twice the number of GBM cells with H19 knockdown experienced apoptosis compared to normal GBM cells under TMZ treatment (Fig. 3a). No difference was found in the number of cells undergoing late apoptosis, and only slightly more H19-deficient cells were found in necrosis in H19-knocked down cells (Fig. 3a). However, with longer treatment or a higher dose of TMZ, a greater percentage of cells are likely to be identified in these two stages. By studying metabolic markers in apoptosis, we found increased Bax expression and decreased Bcl-2 expression in GBM cells with H19 knockdown. Bax is a pro-apoptosis protein and is activated by the tumor suppressor p53 (Miyashita and Reed 1995).

In the final part of this study, we found that GBM cell lines U87MG and U251 both had CSC markers CD133, Nanog, Oct4 and Sox2. H19-knocked down cell lines showed significantly reduced expression of these markers. This finding confirms the theory that GBM cells originate from CSCs (Piccirillo et al. 2009), and also suggests that over-expression of H19 might function to transform normal astrocytes into GSCs. However, this possibility still needs further investigation. Previous research identified that over-expression of H19 in CD133+ GBM cells increased their neurosphere formation and tumor growth (Jiang et al. 2015), which showed the importance of H19 in GSCs. Our results further demonstrate the cancer-promoting role of H19 in GSCs and help expand the existing knowledge from CD133+ GBM cells to ones bearing four different stem cell markers. However, our results do not distinguish the GSC populations bearing these markers. In other words, there may be four GSC populations, each expressing one of the four stem cell markers; but there may also be populations expressing a combination of these four stem cell markers. Further studies on H19 expression and its function on these distinguished GSC populations should prove beneficial. Moreover, the relationship between H19 and the four stem cells markers merits further investigation as they are involved in several important cellular signaling pathways such as PI3K/Akt, p53/Rb and Wnt/β-catenin, malfunction of which usually leads to cancer cell transformation.

The involvement of H19 in maintenance of stem cell markers has been reported in other types of cancer before (Ben-Porath et al. 2008; Gabory et al. 2009; Jiang et al. 2015). Although no detailed mechanisms were shown, we hypothesize that H19 promotes the expression of stem cell markers via regulating the Lin28/Let-7 pathway, which has been shown to maintain stem cell properties in a number of studies. By studying H19 in muscle tissue, where the H19 level is maintained relatively high, a group of researchers found that H19 inhibited Let-7 expression via direct binding, and depletion of H19 accelerated muscle cell differentiation, which suggested a role of H19 in maintaining stem cell properties (Gao et al. 2014). A recent publication reported that suppressing Let-7 increased Oct4 and Sox2 expression in CSC-like oral squamous cell carcinoma (OSCC) by targeting ARID3B and HMGA2, which directly interact with Oct4 and Sox2. The same publication also showed that Lin28 affected the methylation status on Oct4 and Nanog promoter in reprogramming HNOK cells to HNOK-iPSCs (Chien et al. 2015). Although no direct evidence has been found to show the correlation between Let-7 and CD133, several groups reported downregulated Let-7 level in CD133+ positive CSC like cells (Bao et al. 2014; Luo et al. 2013).

Finally, not only did this study reveal the function of H19 in GBM cells, it also added evidence supporting the role of lncRNA in cancer. Besides H19, other lncRNAs have been found to either positively or negatively regulate tumorigenesis and metastasis. Therefore, further studies on lncRNAs and its link to cancer are called for, which could lead to new treatments and perhaps 1 day a cure for cancer.
